# The {332}<113> Twinning Behavior of a Ti-15Mo Medical Alloy during Cyclic Deformation and Its Effect on Microstructure and Performance

**DOI:** 10.3390/ma17071462

**Published:** 2024-03-22

**Authors:** Tiewei Xu, Bingqing Sun, Shanshan Zhang, Yuancai Liu, Wei Sun, Ning Cui, Binjiang Lv

**Affiliations:** School of Mechanical and Automotive Engineering, Qingdao University of Technology, Qingdao 266520, China; twxu@qut.edu.cn (T.X.); sakuna_sun@163.com (B.S.); liuyuancai178@gmail.com (Y.L.); sw290214281@163.com (W.S.); sdcuining@sina.com (N.C.); lbj@qut.edu.cn (B.L.)

**Keywords:** medical titanium alloy, twinning-induced plasticity, {332}<113> twin, cyclic deformation, wear resistance

## Abstract

In this study, the microstructural evolution of a Ti-15Mo medical alloy was investigated, when the in situ cyclic tensile strain had 2% amplitude and the tension–compression cyclic deformation had 1%, 2%, and 3% amplitude. The Vickers hardness and wear resistance of the alloy were also optimized due to the grain-refining effect after cyclic deformation and annealing. The twinning-induced plasticity (TWIP) was considered the main deformation mechanism of the Ti-15Mo alloy during the tensile–compressive cycle deformation with suitable strain amplitude. The {332}<113> twins and boundaries were the main contributors to the grain refinement. The optimal microstructure, hardness, and wear resistance were obtained in the alloy deformed by tension–compression cyclic strain with a 3% strain amplitude. The wear resistance of the annealed alloy in Hank’s solution was excellent in contrast to the original Ti-15Mo alloy due to its reasonable microstructure and hardness. It is clear that abundant twins were formed and retained in the coarse grains of the original alloy after cyclic deformation and annealing, which provided the expected refined grains and performance.

## 1. Introduction

In recent years, the increased demand for biomaterials has accelerated with the aging of the global population. A medical implant metal is a special biomaterial that requires a strict trade-off between mechanical properties, biocompatibility, and manufacturability. Titanium alloys are widely used in biomedical contexts due to their high specific strength, excellent corrosion resistance, and low modulus of elasticity [1,2,3,4]. The Ti-15Mo alloy is likely to form a twinning-induced plasticity (TWIP) effect during plastic deformation and to provide an elasticity modulus that is suitable for medical applications [5]. However, the strength of the metastable β-titanium alloys with a single β phase is insufficient compared with that of α+β titanium alloys; this also affects the service life of medical implants [6,7]. Therefore, retaining a low modulus of elasticity and improving strength have become key challenges for the development of novel medical titanium alloys. 

Transformation-induced plasticity (TRIP) [8], TWIP [9], and dislocation glide [10,11] are the primary deformation mechanisms observed in β-titanium alloys. Sun et al. [12] investigated the early deformation behavior of a Ti-12Mo alloy and identified the simultaneous activation of strain/stress-induced phase transition (β to α″) and primary twinning, such as {332}<113> and {112}<111>. {332}<113> twinning, along with the subsequent secondary twinning, dominates the initial stages of the deformation process. This phenomenon aligns with Schmidt’s law, where twinning deformation adheres to Schmidt’s rule, and the activation stress required for the corresponding deformation mechanism is closely related to the grain orientation factor. In metastable β-titanium alloys, if the {332}<113> and {112}<111> twin systems have the same Schmidt factor (SF), then the {332}<113> twins are preferentially activated due to the higher critical shear stress required to initiate {112}<111> twins [13]. As stress increases, secondary twins [14,15], secondary α″ phase transitions [13], and strains dominated by dislocation glide [16] emerge during deformation. In the practical processes whereby β-titanium alloys are deformed, multiple deformation mechanisms are often combined. The {332}<113> twins, characterized by a large angular interface of 50.57°, are representative of metastable β-titanium alloy twins. Twin boundaries formed during the deformation of β-titanium alloys share similarities with large angular interfaces, hindering dislocation motion and enhancing material strength (dynamic Hall–Petch effect) [17,18,19]. By inducing twinning to refine the β-titanium alloy matrix, a nanoscale sub-crystalline structure with a reduced dislocation density and large angular interfaces can be achieved, thereby increasing strength while maintaining a low elastic modulus and high plasticity in the alloy. Utilizing the cyclic deformation-induced twinning mechanism to refine the microstructure presents a potential breakthrough for optimizing metastable β-titanium alloys.

Analyzing the formation of induced twinning forms the basis for microstructure refinement in β-titanium alloys through the utilization of the TWIP effect. The mechanism underlying the formation of {332}<113> twins remains a debated topic. In their investigation of the Ti-12Mo alloy’s deformation behavior, Ken Cho et al. [20] observed the existence of stress-induced α″ phase transitions as nucleation sites for {332}<113> twins, which are subsequently replaced as stress increases. Castany et al. found that the β phase totally transforms into α″ martensite under stress in a Ti-27Nb (at.%) alloy. {332}<113> β twins are thus not formed directly in the β phase but are the result of the reversion of {130}<310> α″ parent twins occurring in martensite under stress [21]. Miyazaki et al. [22] proposed that in the case of deformation in beta-titanium alloys, the geometric coordination within the deformation structure triggers a complex dragging behavior that leads to {332}<113> twin formation. This {332}<113> twinning exhibits the unique characteristics of primary twinning and a twinning crossover, enabling secondary twinning, and even multilevel twinning within primary twinning. The twinned microstructure formed within the alloy plays a crucial role in enhancing strain hardening and improving the material’s mechanical properties. Traditional nanotwins, gradients, and second-order layered nanotwin structures often necessitate a trade-off between strength and ductility, whereas high-order (>2) layered nanotwins have the potential to enhance the overall mechanical properties of the material [23]. The reported gradient nanograin structure of the material effectively suppresses strain localization, resulting in high ductility at elevated strength levels [24].

In a manner similar to magnesium alloys, various types of twin variants (tensile twins and compression twins) intersect and divide grains, refining them under different stress directions [25]. Under tensile–compressive stresses, β-titanium alloys exhibit interchanged positive and negative SF values, leading to the alternate activation of twins. This phenomenon promotes mutual cross-segmentation, thereby refining the microstructure. Compared to unidirectional tensile or compressive stress, deformation under tensile–compressive cyclic stress facilitates the activation of a larger number of twin variants [26]. Lilensten et al. [27] described the deformation mechanism of {332} mechanical twinning and detwinning in the Ti-15Mo alloy during cyclic loading. Consequently, the initial tissue undergoes favorable division and refinement into ultrafine grain structures. Moreover, the single circumferential strain experienced during tensile–compressive cyclic loading is relatively small, effectively suppressing dislocation slip and proliferation. This characteristic contributes to the alloy’s ability to maintain high plasticity and a low elastic modulus.

Tribological experiments conducted on titanium have demonstrated that samples with an ultrafine-grained structure exhibit an abrasive wear mode, which shows improved wear resistance compared to coarse-grained titanium. In contrast, the wear modes observed in coarse-grained titanium include ploughing and wedge formation [28]. These findings suggest that cyclic deformation can improve the material’s wear resistance.

This study is based on the TWIP mechanism, which introduces twin structures within the grains through cyclic deformation. This process leads to an increased volume fraction of grain boundaries, thereby enhancing the resistance to dislocation slip. Consequently, the Ti-15Mo alloy exhibits improved strength, while maintaining a low modulus of elasticity and enhanced wear resistance.

This study proposes a preparation method that can achieve an ultrafine grain structure for medical-grade Ti-15Mo materials, with the aim of optimizing the material’s properties by creating a microstructure that combines plasticity and toughness. This approach involves multiple small deformations using tension–compression bidirectional cyclic loading to prepare a highly twinned ultrafine-grained metal. The stability of the twinned boundaries is enhanced through various heat treatment regimes. The alloy is subjected to mechanical performance tests, including hardness testing and friction experiments, before and after refinement to investigate the influence of different strain amplitudes and heat treatment methods on the wear resistance of the Ti-15Mo alloy. The aim is to provide insights into and methods for the development and optimization of medical-grade Ti-15Mo alloy materials.

## 2. Experimental

### 2.1. Specimen Preparation

**Figure 1:**
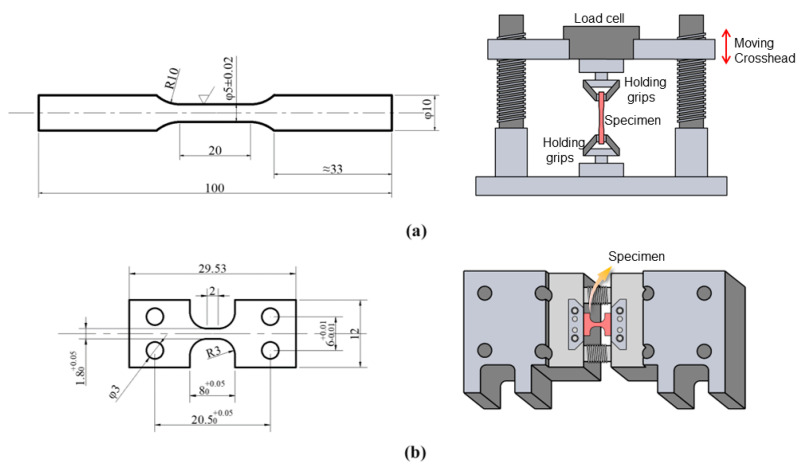
Schematic diagram of specimens (mm): (**a**) tensile and compressive tests; (**b**) in situ EBSD tensile test.

The Ti-15Mo alloy used in this paper was melted with the industrial ingot by vacuum arc melting (VAR) three times. The transformation temperature of the β phase measured by metallography method was approximately 755 °C, and its composition was tested by the chemical analysis method as shown in Table 1. After homogenizing and forging, the alloy was hot rolled to Φ22 mm. Solution treatment (ST) of the samples was conducted in the β phase region at 790 °C for 1 h. The billets were processed with a diameter of 10 mm and a length of 100 mm. The sample for the in situ electron backscatter diffraction (EBSD) tensile test was cut by electrical discharge machining (EDM, DF7745). The parallel section of the in situ tensile sample was polished with 2000-grit sandpaper and subsequently subjected to electrochemical polishing. The electrochemical polishing was performed using an electrolyte solution consisting of perchloric acid, methanol, and n-butanol in a ratio of 1:3:6. A constant current of 2A and a voltage of 35 V were applied during the electrochemical polishing, which lasted for 1 to 2 min. Simultaneously, the environmental temperature was reduced to around zero degrees Celsius using liquid nitrogen. The specimen size for deformation in this paper is illustrated as Figure 1.

### 2.2. In Situ Tensile and Tension–Compression Cyclic Deformation and Heat Treatment

**Figure 2:**
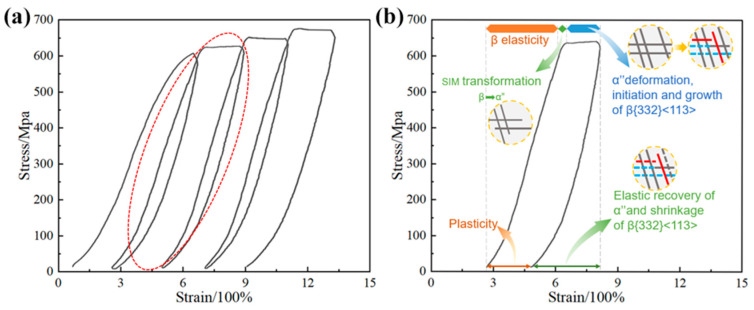
(**a**) Stress–strain curves of the cyclic tensile test with a strain amplitude of 2%; (**b**) the deformation mechanism corresponding to the second cyclic curve.

In situ tensile deformation was conducted with a scanning step size of 0.33 μm and over an area of 1.428 × 1.309 mm. The deformation rate was 0.01 mm/s. The strain amplitude was 2% and cycled 4 times (Figure 2). Cyclic deformation was carried out on INSTRON universal testing machine at 25 °C. Cyclic tension–compression deformation was controlled by the strain amplitude. The amplitude and number of the cyclic deformation with 0.01 s^−1^ strain rate was chosen by ±1% with 1000 times, ±2% with 100 times, and ±3% with 50 times, respectively. After tension–compression cyclic deformation, specimens were cut using EDM, with the observation surface of the samples being parallel to the tensile axis. The resulting cut specimens exhibited a semi-cylindrical shape with a diameter of 6 mm and a length of 5 mm.

The heat treatments of the deformed alloy with 3% strain amplitude were conducted at 730 °C for 7 min, 750 °C for 7 min, and 730 °C for 15 min, with the heating atmosphere being air. The samples were directly placed from room temperature into a preheated furnace set at the desired temperature for a specified period of time, followed by quenching in water. The selection of a temperature range of 730–750 °C for the heat treatment of titanium alloys aims to achieve phase transformation equilibrium, stabilize and preserve the fine-grained microstructure that has been formed, and optimize the material’s performance. The purpose of water quenching is to rapidly cool the material to attain a high hardness state. The high cooling rate provided by water can effectively suppress phase transformations, leading to the development of a metal structure with desirable hardness and strength. The samples after heat treatments were marked as 730-7, 750-7, and 730-15, respectively.

### 2.3. Hardness and Tribological Tests

The Vickers hardness of the original alloy before deformation with coarse grains, the deformed alloy with 3% strain amplitude, and the heat-treated alloy were measured by a micro-Vickers hardness tester (HV-1000, Xi’an, China). The hardness tester was loaded with 500 g for 5 s and measured 10 times to obtain an average value.

Tribological tests were conducted at an ambient temperature using Bruker’s universal mechanical tester (UMT, Billerica, MA, USA). Prior to testing, the specimen underwent mechanical grinding and polishing processes to achieve a mirror-like surface finish. Subsequently, the sample was immersed in Hank’s solution, which was manufactured by Labcoms^TM^ LIFE SCIENCES (Shanghai, China) and served as the friction medium during the tests. The applied load was 5 N, the frequency was set at 1 Hz, and a stroke length of 10 mm was used. The duration of the test was 30 min. A G15 steel ball with a diameter of 9 mm was employed as the friction pair. Following the wear test, the wear surface profile was assessed using a three-dimensional (3D) surface profiler (Qingdao, China). To ensure the reliability of the results, three replicate wear tests were conducted for each microstructure under identical conditions, and the reported wear quantities represent the average values obtained from these tests. A JSM-7800F scanning electron microscope (SEM, Tokyo, Japan) was used to observe the morphology of the friction traces and debris.

## 3. Results and Discussion

### 3.1. In Situ EBSD Tensile Cyclic Deformation with a 2% Strain Amplitude

**Figure 3:**
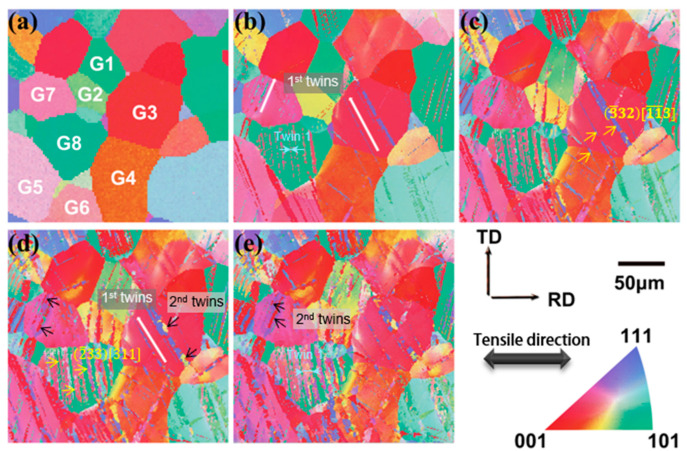
Orientation image maps (OIMs) of (**a**) the initial grains before deformation, (**b**) the grains after the first cyclic deformation, (**c**) the grains after the second cyclic deformation, (**d**) the grains after the third cyclic deformation, and (**e**) the grains after the fourth cyclic deformation.

Under cyclic deformation with a 2% strain amplitude, the stress–strain curve of the Ti-15Mo alloy exhibits notable characteristics. In the initial cycle, no discernible stress plateau is observed, indicating limited plastic deformation. However, in the subsequent three cycles, distinct and pronounced stress plateaus emerge, as evidenced by the microstructural analysis shown in Figure 3. This result signifies the significant contribution of TWIP during the deformation process [29]. TWIP, which is driven by the activation and propagation of twins, plays a pivotal role in enhancing the plasticity and deformation accommodation of the alloy. The appearance of stress plateaus typically corresponds to the plastic deformation stage, which, in this experiment, corresponds to the formation of twinned microstructures [30]. These twinned structures redistribute stress and enable the material to withstand external loads more effectively. This behavior underscores the influence of TWIP on the deformation characteristics of the Ti-15Mo alloy under cyclic loading conditions.

A low-flow stress zone, shown in Figure 2b, first appears in the second cycle of deformation; this is caused by the elastic deformation of the β phase. The flow stress begins to increase to an approximately linear state as the strain increases, with each cycle conforming to this pattern, and a linear increase in strain begins to occur at almost the same stress level in each cycle. This phenomenon occurs because, during the first compression cycle, almost all of the plastic strain is carried by the {332}<113> twins [31]. During cyclic tensile loading, α″ martensite begins to form when the stress exceeds the low-flow stress zone. Before reaching the stress plateau of plastic deformation, the stress-induced martensite causes the stress–strain curve to appear nonlinear. With the increase in stress, the stress–strain curve reaches the stress plateau, when most of the martensite transforms into β{332}<113> twins. Another part of the martensite undergoes inverse phase transformation after stress release, and only a very small amount of martensite remains once the deformation has ended. Throughout the cyclic stretching process, plastic deformation is mainly caused by β{332}<113> twins.

**Figure 4:**
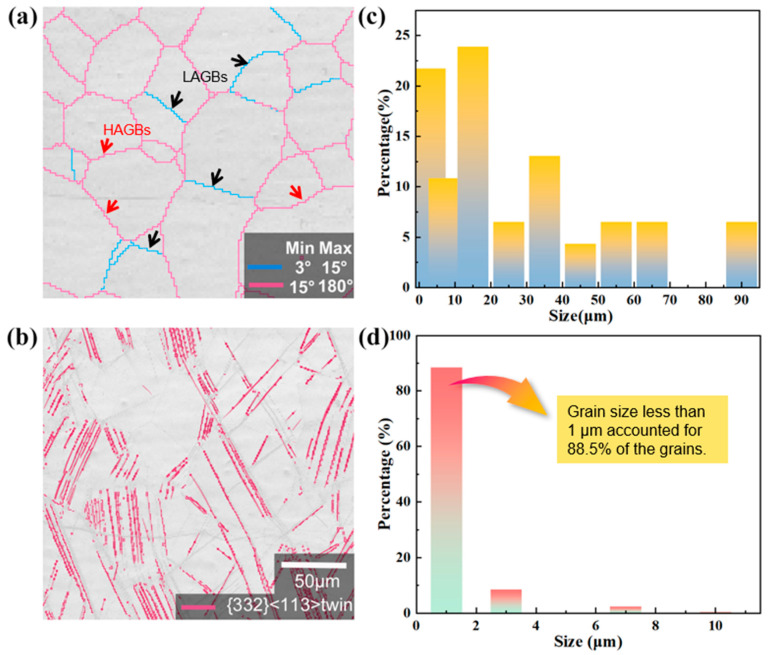
In situ observations of the deformation and graphs of the changes in grain size after in situ tensile deformation with a strain amplitude of 2%: (**a**) grain boundary map, (**b**) {332}<113> twin boundary map, (**c**) grain size statistics before deformation, and (**d**) grain size statistics after deformation.

The in situ EBSD orientation image maps are shown in Figure 3. Multiple twins begin to form within the grain, and as the number of cycles increases, some twins extend through the grain boundaries and into other grains [32]. Subsequently, the secondary twins interact with the primary twins and gradually cover the original grain structure. The nucleation and growth of different types of primary and secondary twins form a complex multiscale twinning network with reduced twin spacing down to the nanometer scale, creating a dynamic Hall–Petch effect. A significant occurrence of multistage twinning is observed in the sample after four cycles, as depicted in Figure 3e. The grain boundary with an angle range of 49.5°–52.5°, highlighted in red by Channel 5 software, corresponds to the grain boundary associated with the {332}<113> twins (Figure 4b). It is evident that the majority of twins formed during deformation are {332}<113> twins. The preferential presence of {332}<113> twins in certain grains can be observed in Figure 3b–e. The selection of grains for twinning has been shown to be influenced by the SF [33,34,35]. Following one cycle of deformation, multiple twins are generated in some grains, occasionally originating from different twinning systems, while other grains remain undeformed (Figure 3b) [36]. The grain boundary angle is illustrated in Figure 4a. From Figure 3b–d, it is apparent that the propagation of twins between grains does not occur at high-angle grain boundaries (HAGBs). For instance, in Figure 3c, the twins in G8 do not transfer to G4 and G7. In contrast, mechanical twins readily propagate across low-angle grain boundaries (LAGBs) [36,37], represented by the color blue in Figure 4a. Twin transfer is observed between G1 and G2, G3 and G4, and G5 and G6 in Figure 3c.

A computational analysis of the critical shear stress required to initiate twinning reveals that, in addition to the presence of sufficient forces within the grain, the twinning direction and twinning plane angle must satisfy the critical shear stress conditions. Consequently, grains with softer orientations, which are more conducive to twinning, tend to initiate twinning during the deformation process. On the other hand, grains with harder orientations require larger forces and deformation to initiate twinning. The critical shear stress necessary to initiate twinning is comparable for grains with similar orientations, facilitating the direct continuation of the twinning boundary. As deformation progresses, twinning multiplies within the grains. However, the deformation remains inhomogeneous due to the varying inclination of the oriented grains towards twinning deformation.

The impact of twinning on grain characteristics is revealed in Figure 4c,d, where the grain size statistics before and after deformation based on grain boundaries are presented using Channel 5 software. It is evident that a significant grain refinement occurs, with the grain size of the alloy usually measuring less than 1 μm following cyclic deformation. The deformation processes during the tensile and compression cycles induce changes in the distribution of the energy stored within the alloy, with stored energy playing a pivotal role in grain nucleation and growth [37].

**Figure 5:**
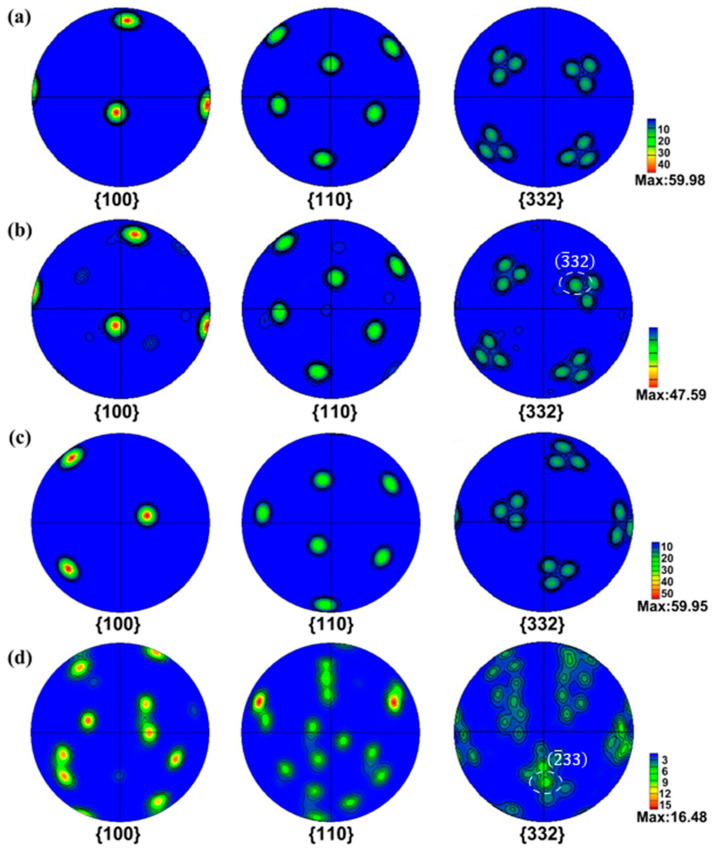
Pole figures (PFs) of Grain 3 and Grain 8 before and after the in situ cyclic tensile test: (**a**) G3 before the in situ cyclic tensile test, (**b**) G3 after the in situ cyclic tensile test, (**c**) G8 before the in situ cyclic tensile test, and (**d**) G8 after the in situ cyclic tensile test.

Figure 5c,d illustrates the intrinsic alterations in the texture of Grain 8 under in situ tensile cyclic deformation. A similar trend is observed in nearly every grain, depicting a reduction in the textural intensity of PFs with increasing stretching cycles, as observed when compared with Figure 3. The reduction in textural intensity causes the material to display more uniform properties in different directions, regardless of specific grain orientations. This reduction in textural intensity enhances the mechanical performance of the material [38]. Based on these findings, it can be inferred that the tensile cycles contribute to grain refinement and to the reduction in textural intensity in the Ti-15Mo alloy. Polar plots in the {332} direction, as presented in Figure 5b,d, indicate the types of twin variants produced following tensile deformation. Table 2 displays the Schmidt factors corresponding to the 12 potential variants generated in grains G3 and G8. Notably, the Schmidt factor value of the variant produced after deformation is the largest among the Schmidt factors of the 12 variants, highlighting a strong correlation between the production of twin variants and the Schmidt factor. Due to the low critical shear stress required for growth, {332}<113> twins emerge as the predominant twinning mode in Ti-15Mo alloys.

**Figure 6:**
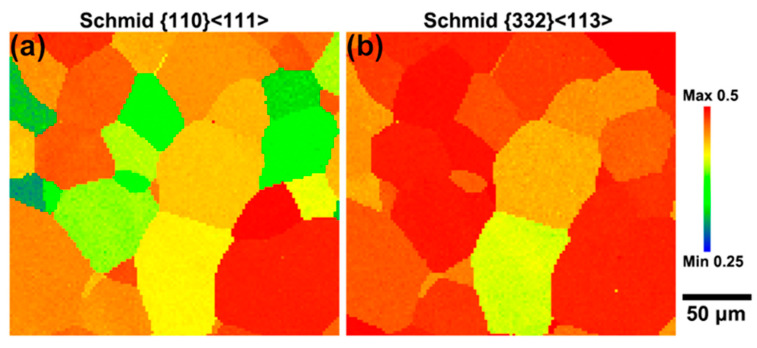
Schmidt factor distribution maps for the Ti-15Mo alloy after one cycle of deformation in the slip system: (**a**) {110}<111> and (**b**) {332}<113> twins.

Figure 6 illustrates the SF for the slip system {110}<111> and the {332}<113> twins. Remarkably, the SF of the {332}<113> twins is substantially higher than that of the slip system. Grains with higher SF values tend to activate {332}<113> twinning as their primary deformation mode, rendering them more susceptible to this twinning behavior. The proliferation of twins during deformation is believed to account for the alloy’s elevated and stable strain-hardening rate [38]. The non-uniformity of the deformation mechanism implies that, under a given macroscopic strain, individual grains do not experience identical local strain conditions. Grains that undergo multiple twinning events may undergo rapid local strain hardening, aligning with the concept of the dynamic Hall–Petch mechanism [39,40,41]. Conversely, neighboring grains devoid of twins exhibit lower levels of local strain hardening.

### 3.2. Cyclic Tension–Compression Tests with 1%, 2%, and 3% Strain Amplitude

The microstructural changes in the β-titanium alloys during tension–compression cyclic deformation are predominantly influenced by factors such as the stress amplitude, frequency, and the overall number of cycles [16,30]. It is worth noting that the stress direction plays a crucial role in determining the type, quantity, and size of the twin variants. Therefore, the deliberate utilization of tension–compression cyclic deformation is revealed to be an attractive avenue for promoting the nucleation of twin variants, as well as facilitating cross-segmentation within β-Ti alloys.

**Figure 7:**
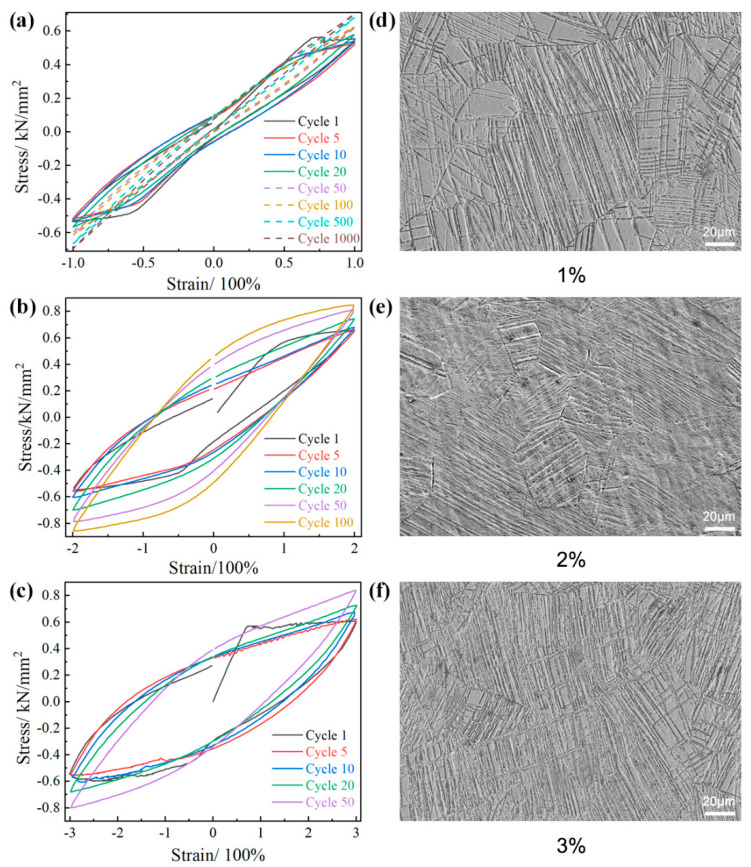
(**a**) Hysteresis curve at 1% strain, (**b**) 2% strain, and (**c**) 3% strain; (**d**) SEM image at 1% strain, (**e**) 2% strain, and (**f**) 3% strain.

The stress–strain curves obtained from cyclic deformation experiments (Figure 7a–c) demonstrate that the area enclosed by the cyclic curve increases as the strain amplitude increases. However, it is notable that the increase in area is more significant for strain amplitudes ranging from 1% to 2% in comparison to those between 2% and 3%. This observation indicates that the alloy’s energy absorption reaches its maximum at a strain amplitude of 3%, indicating an optimal range for controlling and optimizing the alloy’s microstructure through deformation. The mechanical response of Ti-15Mo under cyclic deformation with a 1% strain amplitude is shown in Figure 7a. The shape and slope of the hysteresis curve vary with the number of cycles. In the initial cycles, the hysteresis curve is wide, and the magnitude of peak and valley stress values is relatively low, with relatively flat upper and lower segments. As the number of cycles increases, the hysteresis curve becomes slender and sharp, and the overall slope of the hysteresis curve tends to increase. The area enclosed by the cyclic curve indicates that the alloy undergoes cyclic hardening after 50 cycles of deformation. For the alloy with a 2% strain amplitude (Figure 7b), it is still in the strain-hardening stage after 50 cycles and has not reached cyclic stability. The stress–strain curve for the sample with a 3% strain amplitude (Figure 7c) exhibits a sawtooth pattern at the beginning of each cycle, which is attributed to phase transformations. The hysteresis curve shows a similar trend for all three strain amplitudes, transitioning from a wide to a narrow shape as the number of cycles increases. Moreover, the magnitude of peak and valley stress values increases, and the overall slope of the hysteresis curve tends to rise. This indicates that Ti-15Mo possesses significant cyclic hardening capacity at each strain amplitude, with a stronger cyclic hardening observed for larger and faster strain amplitude increments.

The initial cycle of tensile deformation exhibits both elastic and plastic behavior for all three strain amplitudes, as evident from the shapes of the stress–strain curves in Figure 7a–c. In the initial cycle, the alloy experiences a combination of elastic and plastic deformation, and this behavior is observed across the different strain amplitudes. During the subsequent tension–compression cycles, the specimen displays plastic deformation during the compression phase, while elastic deformation occurs at the beginning and end of each cycle. This suggests the presence of certain obstacles that hinder the reversible motion of the twin interface during cyclic deformation.

Two potential factors contributing to this phenomenon can be considered: First, {332}<113> twinning is associated with a significant number of dislocations at the twin boundary, and these dislocations undergo movement and entanglement during cyclic deformation. The entangled dislocations and the occurrence of secondary twinning act as hindrances to the reversible motion of the twin interface [42]. Second, stress-induced martensite formation, followed by reverse phase transformation, may occur during cyclic deformation. The irregular shape of the hysteresis curve can be attributed to the reversible motion of α″ martensite induced by changes in the stress direction, similar to the detwinning behavior of {332}<113> twins [30]. SEM images of the samples deformed by tension–compression cycles at 1%, 2%, and 3% strain amplitudes are presented in Figure 7d–f. The analysis of the deformation mechanism and stress–strain curve of the β-titanium alloy under tension–compression cyclic deformation conditions confirms the generation of numerous twin crystals. As anticipated, Figure 7d–f shows the production of a substantial quantity of twins in the deformed samples. Samples subjected to a 3% strain amplitude exhibit a high density and large number of twins within the grains. The original grains are almost entirely filled with twins, resulting in their subdivision into smaller sizes and the development of a refined microstructure. These twin formations contribute to the enhancement of the properties of the β-titanium alloy.

### 3.3. Effect of Heat Treatment on the Hardness and Tribological Properties of the Alloy

**Figure 8:**
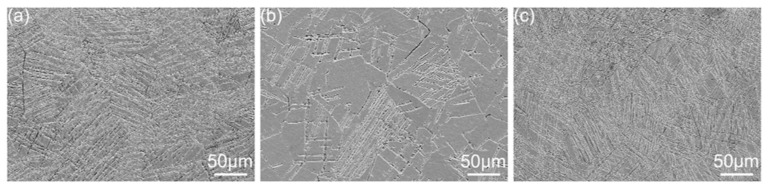
SEM images of the Ti-15Mo alloy with 3% strain amplitude after three heat treatments: (**a**) 730-7, (**b**) 750-7, and (**c**) 730-15.

Severe plastic deformation often results in high residual stress, which has a significant effect on the comprehensive performance and practical application of the alloy. Heat treatment is necessary to alleviate the residual stress. In this study, three different heat treatment processes were performed on the 3% cyclically deformed samples, and the SEM microstructures of the heat-treated samples are presented in Figure 8. It is evident that the number of twins in the sample subjected to heat treatment at 730 °C differs significantly from that in the 3% deformed sample. Moreover, the sample heated at 750 °C for seven minutes exhibits a considerably lower number of twins compared to the other two heat treatment conditions. This discrepancy can be attributed to the chosen heat treatment temperature being excessively high to meet the thermodynamic conditions required for recrystallization [43]. During the heat treatment, complete recovery, recrystallization, and grain growth take place, leading to a decrease in the twin density within the alloy.

**Figure 9:**
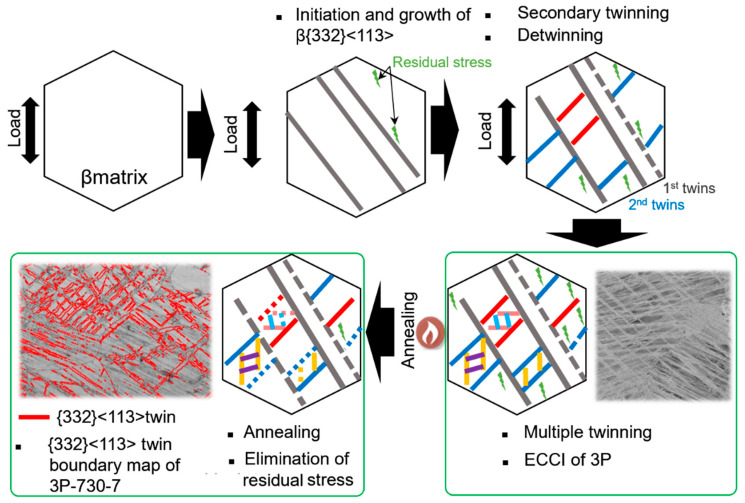
Schematic diagram of the formation of {332} twins in the Ti-15Mo alloy after deformation and the annealing treatment and the OIM of the {332}<113> twin boundary map of 730-7. (ECCI: electron channeling contrast imaging; 3P: 3% deformed sample; 3P-730-7: annealing performed at 730 °C for 7 min in the 3% deformed sample).

The formation of {332} twins in the Ti-15Mo alloy after deformation and the annealing treatment is depicted in Figure 9. Additionally, the twin boundaries (49.5°–52.5°) of the {332}<113> twins within the 730-7 sample are highlighted in red in Figure 9, illustrating that {332}<113> twins dominate the twin type within the sample after the heat treatment.

**Figure 10:**
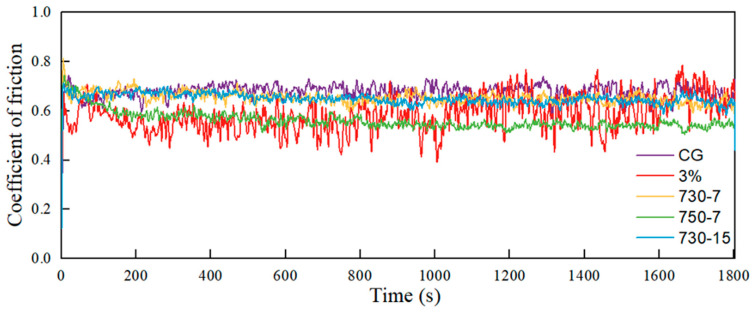
The friction curves of the CG, 3% deformation, and heat-treated samples.

**Figure 11:**
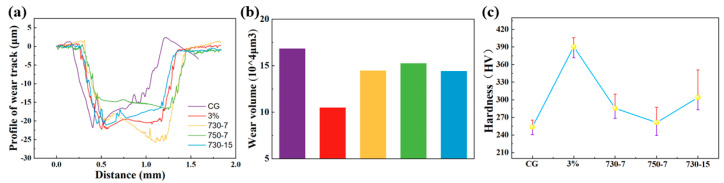
(**a**) Chart of the profiles of wear track, (**b**) wear volume statistics, and (**c**) Vickers hardness.

The friction behavior of the CG, 3% deformation, and heat-treated samples is presented in Figure 10. It is evident that, other than the 3% deformed sample, all of the samples exhibit a relatively stable friction process without significant fluctuations. The wear resistance of an alloy is strongly influenced by its hardness [44,45]. The 3% strain amplitude sample possesses high levels of hardness, resulting in an increased resistance to shear deformation during the friction process (shown as Figure 11c). Consequently, it becomes more difficult for the friction process to proceed smoothly, leading to pronounced fluctuations in the friction curve.

Figure 11 presents the characteristic cross-sectional profile along with the wear volume and Vickers hardness variations for the coarse-grained (CG), 3% deformed, and heat-treated samples. Notably, distinct differences in the wear trajectories between the various samples are evident in Figure 11a. During the process of rubbing against the substrate, the surface undergoes detachment under stress, leaving behind relatively hard particles that continue to impact the substrate surface, thereby generating plow grooves due to cyclic stress. Concurrently, material buildup occurs on both sides of the wear track due to shear forces.

Based on Figure 11b, it can be observed that the 3% deformed sample exhibits the lowest wear volume, while the CG sample displays the highest wear volume. The wear of the heat-treated samples, although relatively high compared to the 3% deformed sample, remains lower than that of the CG sample. This observation establishes a positive correlation between wear resistance and sample hardness. Cyclic tensile deformation not only significantly increases the hardness of the alloy but also exerts a crucial positive influence on its wear resistance.

The Vickers hardness results, which are shown in Figure 11c, reveal a substantial increase in Vickers hardness from 253.64 HV to 390.50 HV for the sample subjected to 3% cyclic deformation. This considerable enhancement of hardness can be attributed to the pronounced occurrence of twinning and the presence of residual stress resulting from the cyclic tension and compression deformation, contributing to the effective hardening of the alloy. However, following different heat treatments, the Vickers hardness of the samples exhibits varying degrees of reduction compared to that of the 3% deformed samples. This reduction in hardness is associated with the elimination of residual stress and the decrease in twin density. Notably, the abnormal grain growth observed in sample 750-7 leads to a significant reduction in the alloy’s hardness.

**Figure 12:**
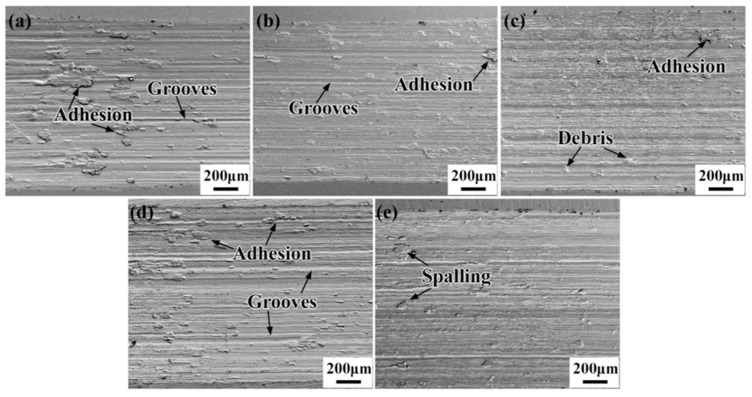
Wear morphology of the (**a**) CG, (**b**) 3% deformed, (**c**) 730-7, (**d**) 750-7, and (**e**) 730-15 samples.

The wear surface morphology of the CG, 3% deformed, and heat-treated samples is presented in Figure 12. Under identical loading conditions, the wear scratches on all five samples appear relatively flat, exhibiting surface damage resulting from micro-cutting, delamination, and spalling. Plowing and micro-cutting give rise to the formation of numerous parallel grooves. It is worth noting that the depth of these grooves exhibits a clear correlation with the hardness of the samples, with shallower grooves observed in the wear marks of harder samples [46]. Fatigue wear occurs when contact stresses momentarily surpass the yield strength of the Ti-15Mo alloy being tested. The development of lamellar cracks arises from the repeated work hardening of the wear surface during reciprocal sliding. As these cracks propagate and expand, they eventually lead to the lamellar cracking of the surface material [46,47].

From Figure 12a,d, it is evident that the size of wear marks on the CG sample and the 750-7 sample is significantly larger compared to the other samples. These samples exhibit deep surface plow grooves, with larger-sized abrasive chips predominantly adhering to the surface. The presence of numerous abrasive chips reveals the adhesive wear. These chips continuously compact and weld to the sample surface during reciprocal friction, resulting in adhesive wear. In contrast, the samples subjected to 3% deformation, namely, the 730-7 and 730-15 samples, display fewer and smaller damage marks compared to the two samples with lower levels of hardness. As depicted in Figure 12b,c,e, the debris size is small and non-adherent, with some smaller abrasive particles distributed on the surface. The dominant wear mechanism in these cases is abrasive wear. Overall, the samples with 3% deformation, particularly the 730-7 and 730-15 specimens, exhibit reduced damage in terms of the number and size of wear marks compared to the samples with lower hardness. The presence of smaller, non-adherent debris and the distribution of smaller abrasive particles on the surface further support the predominance of abrasive wear as the primary wear mechanism.

## 4. Conclusions

The microstructural evolution of the Ti-15Mo alloy was studied via an in situ tensile test and three kinds of tension–compression cyclic deformation tests with different strain amplitudes. We investigated the effects of different microstructures and heat treatment procedures on the alloy’s Vickers hardness and wear resistance. The main conclusions are as follows.

1. Cyclical tensile strain is beneficial to refining grains and improving the microstructure of the Ti-15Mo alloy. The TWIP effect induced by {332}<113> twins is the primary mechanism for grain refinement.
2. In contrast to the alloy produced with 1% and 2% tension–compression cyclic deformation, the alloy deformed with a 3% strain amplitude had the optimal microstructure due to abundant and crossed twins.
3. The hardness and friction properties of Ti-15Mo alloys in variable forms after undergoing different heat treatments show large differences, and the selection of a suitable heat treatment regime can further optimize the properties of the alloy.

## Figures and Tables

**Table 1:** Chemical composition and phase transformation temperatures of the β phase in the Ti-15Mo alloy.

Element	Ti	Mo	O
Mass fraction/%	85.22	14.7	0.08
Temperature, A_f_/°C		755	

**Table 2:** SF values of the twelve possible twin variants in G3 and G8.

	$\left(332)[11\bar{3}\right]$	$\left(323\right)[1\bar{3}1]$	$\left(233\right)[\bar{3}11]$	$\left(\bar{3}32\right)[\bar{1}\bar{1}3]$	$\left(\bar{3}23\right)[\bar{1}\bar{3}1]$	$\left(\bar{2}33\right)[311]$	$\left(3\bar{3}2\right)[1\bar{1}\bar{3}]$	$\left(3\bar{2}3\right)[131]$	$\left(2\bar{3}3\right)[\bar{3}\bar{1}1]$	$\left(\bar{3}\bar{3}2\right)[113]$	$\left(\bar{3}\bar{2}3\right)[\bar{1}31]$	$\left(\bar{2}\bar{3}3\right)[3\bar{1}1]$
**G3**	−0.4965	0.1641	0.0533	0.2257	0	0.3693	−0.3734	0.3939	−0.0369	0.0657	0.1723	0.1641
**G8**	−0.4154	−0.1929	0.3709	0.4153	−0.1926	0.3709	0	0.2225	0.0148	0	0.2225	0.0148

## Data Availability

Data are contained within the article.
